# An Innovative Use of the QuEChERs Method and LC-MS/MS Technique for Fast and Simple Determination of Quinolizidine Alkaloids in Leguminous Plants

**DOI:** 10.3390/molecules30204085

**Published:** 2025-10-14

**Authors:** Ewa Rutkowska

**Affiliations:** Institute of Plant Protection—National Research Institute, Chełmońskiego 22 St, 15-195 Białystok, Poland; e.rutkowska@iorpib.poznan.pl

**Keywords:** quinolizidine alkaloids, leguminous plants, LC-MS/MS, QuEChERS, analytical method, optimisation

## Abstract

Quinolizidine alkaloids, found particularly in leguminous plants (*Fabaceae*), are known for their role in plant protection, acting as toxic secondary metabolites against pests and pathogens. However, their toxicity also makes them anti-nutritional factors in food and feed. Therefore, it is necessary to monitor their presence. The aim of this study is to optimise two stages of the research procedure, i.e., (1) the conditions of LC–MS/MS instrumental analysis for the simultaneous determination of five alkaloids: angustifolin, hydroxylupanine, sparteine, and two geometric isomers of lupanine and isolupanine, and (2) the extraction and isolation stage of six different leguminous matrices: field beans, peas, lupins (narrow-leaved, white, yellow) and lentils. The modified and validated QuEChERS method based on LC-MS/MS shows acceptable recoveries (71–115%) with relative standard deviation <15%. A slight matrix effect (−20–14%) was observed. The uncertainty of the method <28%. The developed method shows significant progress in terms of sensitivity, achieving a detection limit as low as 0.01 mg/kg. This is a significant improvement over existing analytical methods and highlights the great potential of this method for detecting trace amounts. The innovative, sensitive, and selective method, offering simplicity and speed, was applied to the analysis of real leguminous samples.

## 1. Introduction

Leguminous plants (*Fabaceae*) are a significant part of many people’s diets. The most common large-grain legumes include broad bean, chickpea, field pea, lentil, soybean, lupins (narrow-leaved, white, yellow), field bean, vetches (hairy, common) and small-grain legumes: clover (white, alsike, Persian, red), sand lucerne, and white melilot [1]. They are an important source of protein, fibre, B vitamins, iron, calcium, magnesium, potassium, zinc and selenium, rendering them a significant part of the human diet. They have an important role in the prevention of cardiovascular disease, type 2 diabetes, obesity and certain types of cancer. Furthermore, legumes are a crucial component of vegetarian and vegan diets, as they can serve as a substitute for animal protein, ensuring the proper function of the body [2]. In addition to essential and valuable nutrients and energy sources, they may contain toxic substances produced during cultivation, harvesting, processing, storage and transport. The toxicity of leguminous plants is attributed to naturally occurring toxins, particularly alkaloids [3].

Alkaloids are defined as nitrogen-containing organic compounds that can produce pronounced physiological responses, excluding amino acids, nucleic acids and certain natural nitrogen-containing compounds (tetracycline, kanamycin). They could be produced as secondary metabolites by various organisms including bacteria, fungi and plants, which exhibit useful bioactivities [4]. In plant species, they are produced as a defense mechanism against insect pests, pathogens, herbivorous animals, and competing plants due to their typical bitter taste and toxic effects [5].

Over 16,000 alkaloids are known, including tropane alkaloids (TAs), quinoline alkaloids, isoquinoline alkaloids, quinolizidine alkaloids (QAs), indole alkaloids, pyrrolizidine alkaloids (PAs), purine alkaloids, imidazole alkaloids, pyridine alkaloids and piperidine alkaloids [6]. Pyrrolizidine alkaloids are mainly found in black and green tea leaves and herbs [7], while tropane alkaloids are present in plant-based products such as flour, cereals, biscuits, cakes, bread, pasta, herbal teas and legumes [8]. Quinolizidine alkaloids are the most common and toxicologically significant alkaloids in leguminous plants. The main alkaloids of this group are angustifoline, sparteine, isolupanine, hydroxylupanine and lupanine [9]. In humans, QAs can inhibit acetylcholine receptors in both the central nervous system and the peripheral autonomic nervous system, which can cause respiratory failure and ultimately death. Acute poisoning with QAs presents as malaise, nausea, respiratory arrest, weakness and coma [10]. Despite this, the European Union (EU) has established maximum residue limits (MRLs) only for specific alkaloids (atropine, morphine, codeine, scopolamine, ergot and 21 PAs expressed as a sum) in selected matrices (cereals and cereal products, tea, dried herbs, poppy seeds and poppy seed products) [11].

It is essential to accurately determine their content in plant material and food products due to their potential toxic effects [7]. To ensure high quality of leguminous plants, and due to the low content of certain QAs, selective and highly sensitive analytical methods should be used to monitor the levels of undesirable QAs. The problem with analysing QAs is due to their low water content and the presence of a number of interfering compounds (proteins, sugars and fats) that negatively affect the sensitivity and selectivity of the method [12]. Liquid chromatography with tandem mass spectrometry (LC–MS/MS) is the most commonly used method for the identification and quantitative determination of QAs [5,12,13,14], less commonly ultra-high performance liquid chromatography coupled with quadrupole time-of-flight mass spectrometry (UHPLC–QTOF–MS) [15], gas chromatography (GC) with mass spectrometry (MS) [16] or with a flame ionisation detector (FID) [17].

The most widely used methods for isolating QAs involve extraction by dissolution in an acidic solution, followed by alkalisation and further purification by liquid–liquid or solid-phase extraction [9,14,15,16,17] and solid–liquid extraction [5], which is labour-intensive, time-consuming and requires large quantities of solvents. The QuEChERS (quick, easy, cheap, effective, rugged, and safe) method is a commonly employed analytical procedure for preparing various matrices for testing [18], such as fruit, vegetables [19], bees and bee products [20], oilseeds and oilseed products [21], cereals and cereal products [22], herbs [23], and dairy products [24]. It is also used to determine the residues of a wide range of analytes, such as pesticides [19,25], mycotoxins [26], polycyclic aromatic hydrocarbons (PAHs) [27] and pharmaceuticals [28]. This method is notable for its speed and simplicity in preparing samples for testing, meets the advantages of green chemistry and offers good repeatability and accuracy of determinations, while enabling the simultaneous determination of multiple compounds in various matrices. Due to the growing demand for analysis of quinolizidine alkaloids, the possibility of adapting this method to QA determination in leguminous plants was considered. Only a few studies in the literature describe the use of this procedure for QA analysis, focusing mainly on different lupin species, and the limits of quantification (LOQ) are high (0.55–1 mg/kg) [10,14]. However, no research has been conducted on legumes such as peas, lentils or soybeans.

This study sought to develop a universal, effective and sensitive analytical method for the simultaneous determination of five quinolizidine alkaloids: hydroxylupanine, angustifoline, sparteine, including geometric isomers of lupanine and isolupanine in diverse matrices of legume seeds (field beans, peas, white lupins, yellow lupins, narrow-leaved lupins, lentils with low water content and high protein content. As part of the research, the QuEChERS method was modified and optimised, and appropriate conditions for separation and quantification using LC–MS/MS were selected. This method was employed to analyse actual samples of leguminous plants.

## 2. Results and Discussion

### 2.1. QuEChERS Method and LC-MS/MS Techniques Development

**Figure 1:**
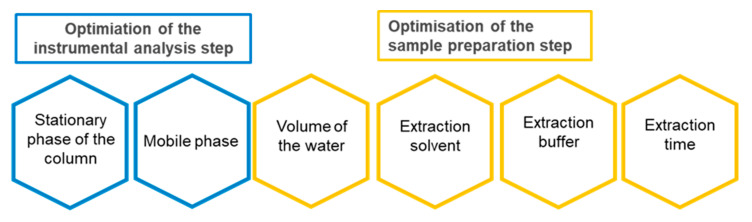
Two-step optimisation of the quinolizidine alkaloids determination method.

In the course of research, key stages of sample preparation for testing complex matrices of legume seeds with low water content and high protein content were optimised, and conditions for instrumental analysis were developed (Figure 1). The following factors were analysed: stationary and mobile phases, water volume, type of extraction solvent and buffer, and extraction time (Supplementary Table S1). The parameter used to assess the suitability of the procedure was recovery (Supplementary Table S2).

In the first experimental stage, the parameters of tandem mass spectrometry detection were optimised by direct injection of each standard analyte (10 µg/mL) at a flow rate of 5 μL/min. For each alkaloid, the two most intense multiple reaction monitoring (MRM) transitions were selected based on the signal-to-noise (S/N) ratio and defined as a quantifier (precursor ion) and qualifier (confirmation ion).

To achieve good separation of analytes and obtain acceptable peak shapes, minimise the effects of fronting and tailing, which interfere with the correct quantification of analytes, and to achieve the highest possible sensitivity, a series of injections was performed, and different stationary and mobile phases were tested simultaneously.

**Figure 2:**
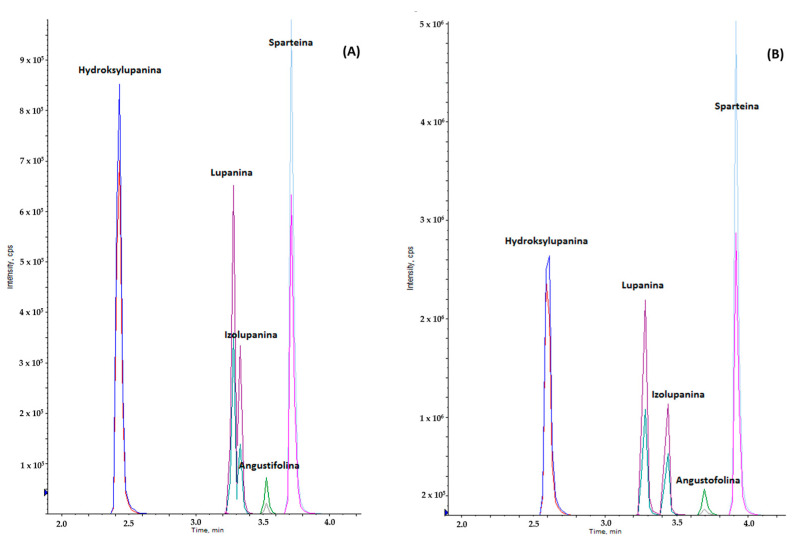
Chromatogram of a mixture of five quinolizidine alkaloids with a concentration of 0.5 mg/l (**A**) stationary phase C18, phase A: water with 0.1% acetic acid, phase B: methanol with 0.1% acetic acid, the elution gradient A%:B%, 95:5, 5:95, 95:5 (**B**) stationary phase HILIC, phase A: water with 0.1% acetic acid, phase B: acetonitrile with 0.1% acetic acid, the elution gradient A%:B%, 5:95, 95:5, 5:95.

Alkaloids are moderately polar, basic organic compounds with a complex structure [29], so the influence of stationary phases was assessed using a hydrophobic (non-polar) octadecylsilane (C18) ligand (Phenomenex Kinetex XB C18, 2.6 μm; 100 × 2.1 mm) and a HILIC (polar) system (often silica gel, diol, amide, NH_2_ or CN) (Kinetex HILIC, 1.7 μm; 50 × 2.1 mm and 1.7 μm; 100 × 2.1 mm; Phenomenex, Torrance, CA, USA). The use of the C18 stationary phase resulted in good separation of analytes, except for the geometric isomers of lupanine and isolupanine, where co-elution occurred. Where HILIC was employed as the stationary phase, better retention of all alkaloids was observed, as well as partial separation of the analytical signals of lupanine and isolupanine. To achieve complete separation of the chromatographic peaks of these alkaloids, a column twice as long (Phenomenex Kinetex HILIC 1.7 μm; 100 × 2.1 mm) was used (Figure 2). HILIC offers significant mechanical advantages in the separation of polar compounds that are highly soluble in the aqueous mobile phase, such as lupanine and isolupanine, compared to C18 columns, as it uses a hydrophilic stationary phase that interacts with water, creating a distinct, highly viscous water-rich layer on the surface where analytes are retained through separation and polar interactions. The separation mechanism involves the separation of analytes between the mobile phase and the water-rich layer of the stationary phase. The increasing water content gradient in the mobile phase then causes selective elution of more polar analytes. C18 columns rely on hydrophobic interactions between nonpolar stationary phases and nonpolar analytes [30]. Lupanine and isolupanine are not sufficiently hydrophobic to be retained by the C18 stationary phase, resulting in poor retention and low resolution.

Several combinations of mobile phases were tested: water, methanol and acetonitrile, compounds with different polarities, and an acid modifier was added to enhance ionisation: acetic acid and formic acid. Satisfactory results were obtained using water with 0.1% acetic acid as phase A and acetonitrile as phase B, but tailing was noted in the case of sparteine. The solution to this problem was to use 0.1% formic acid instead of acetic acid, which resulted in effective chromatographic separation (peak shape) and method sensitivity (ionisation efficiency).

Nadmar et al. [10] used an LC–MS/MS system to separate 15 QAs, including those identified in this study, on a column with an octadecyl stationary phase (1.7 μm, 100 × 2.1 mm), using mobile phases such as (A) ammonium carbonate buffer and (B) methanol; however, the determined limit of quantification (1 mg/kg) was 100 times higher than that obtained in our study (0.01 mg/kg).

A new approach to the determination of multi-component pesticide residues in matrices with high moisture content and low fat content (fruit and vegetables) was introduced by Anastassiades et al. [18], which was based on extraction with acetonitrile and dispersive solid-phase extraction as part of purification (without buffering). Researchers have frequently modified this original method to suit different groups of compounds and types of samples. The most common modifications involved the use of acetate buffer [31] or citrate buffer [32]. The modifications also included the addition of water to ensure effective extraction of compounds and separation of the organic phase from the aqueous phase; selection of salts for better separation of analytes from interfering compounds; selection of sorbent at the purification stage to remove substances interfering with the analytes studied; change in buffers to obtain the required pH for the analytes; inclusion of a freezing step to remove fats from the samples [26,28].

Due to the flexibility of the QuEChERS method, attempts were made in the described studies to modify it for the determination of alkaloids in the legume matrix. Since alkaloids have different chemical properties than pesticides (higher polarity, alkalinity), the original protocol needed to be modified. Different buffers were employed to maintain the appropriate pH and limit the decomposition or loss of alkaloids. Water was added to improve the extraction efficiency of leguminous plants with low water content. Solvents with different polarities were also tested [33].

The first parameter tested to optimise the method was the addition of water. For this purpose, experiments were carried out using 3, 6, 9, and 12 mL of water. When 3 and 6 mL of water were applied, it was completely absorbed. In comparison, the use of both 9 and 12 mL of water yielded satisfactory parameters that did not differ significantly at all stages of the analysis for all tested matrices. The addition of 9 mL of water was chosen for further optimisation.

The next experimental step was to select a suitable extracting agent, which should have a high solubility of the tested analytes, while minimising the impact of co-extracted matrix components [34]. Solvents with different polarities were used in the experiments, namely ethyl acetate, methanol, acetonitrile and water with the optional addition of formic acid, which improves the stability of basic compounds [35]. The use of 10 mL of acetonitrile as an extractant resulted in the highest process yield. When ethyl acetate, methanol or water were applied, the recovery rates were 25–50% lower compared to acetonitrile. The addition of formic acid did not affect the extraction efficiency in any case. The effectiveness of acetonitrile and methanol with the addition of formic acid as extraction solvents for determining QAs in lupin samples has been evaluated by other researchers [5,10]. Hwang et al. [12] used UPLC–MS/MS to identify five QAs in lupin using methanol with 1% formic acid as the extraction solvent. On the other hand, Eugelio et al. [5] developed a method for determining 13 QAs in lupin using methanol extraction with 1% formic acid and purification by solid-phase extraction (SPE), followed by analysis by HPLC–MS/MS. However, the determined compounds did not include isolupanin, one of the main QAs present in lupin [5,12,17].

To monitor 15 QAs in lupin, Nadmar et al. [10] developed and validated a determination method based on methanol: water extraction, followed by purification on SPE (C18 phase) and analysis with LC–MS/MS. However, the method developed and validated by Khedr et al. [14] allowed for the analysis of five QAs in seeds of various lupin species, which included extraction with an ultrasonically assisted water/acetonitrile mixture, followed by separation with NaCl and MgSO_4_ and alkalisation in the presence of NaOH, and then analysis by LC–MS/MS. Both methods examined all major QAs present in lupin, but a relatively high limit of quantification (LOQ) was obtained (0.55, 1 mg/kg) [10,14].

At the same time, the influence of buffers (citrate, acetate, borate) and the optimal extraction time (5 and 10 min) were analysed. The best results were achieved using extraction in the presence of citrate buffer at 10 min. There are no research papers reporting experiments with different buffers in the determination of alkaloids in legume seeds.

### 2.2. Method Validation Results

The validation parameters obtained confirmed that the chromatographic conditions and sample preparation for testing were selected correctly.

The recoveries for all tested alkaloids were within the range of 70–120% with a relative standard deviation of less than 20%. In the analysed concentration range of 0.01–10.0 µg/mL, satisfactory linearity of the method was obtained, confirmed by a correlation coefficient of R^2^ > 0.999. Analyes of interest showed accetable values (from −14 to 18%) of back-calculted concentration determined at seven different concentrations. The matrix effect did not significantly affect signal suppression or enhancement and ranged from −19 to 14%. Although the matrix effect is within SANTE guidelines [36], there is a tendency for the signal of the analytes being analyzed to be suppressed rather than amplified, which is characteristic of LC/MS/MS. This problem usually occurs due to the presence of co-extracted components in the samples. Legume seeds contain large amounts of proteins [2], which can reduce the response of target analytes due to competition for ionization based on the highest charge affinity of different eluting molecules [37]. Similarly, Eugelio et al. [5] observed more frequent signal suppression than enhancement for the alkaloids studied, including angustifolin, hydroxylupanine, lupanine, and sparteine in lupine matrices.

The LOD of the tested compounds was determined to be 0.003 mg/kg, and the LOQ was 0.01 mg/kg. The estimated expanded uncertainty of the entire analytical process did not exceed 30% (coefficient of variation k = 2, confidence level 95%) (Table 1). The developed method is competitive with other previously described methods because it allows QA to be detected at lower levels. In the method developed by Namdar et al. [10], the LOD was 0.1–0.5 mg/kg and the LOQ was 1 mg/kg. Similarly, in the method described by Hwang et al. [12], the LOD was in the range of 0.5–1.7 mg/kg and the LOQ was 1.5–5.7 mg/kg.

### 2.3. Analysis of QAs in Leguminous Plants

An optimised testing procedure, with appropriately selected QuEChERS method parameters and LC–MS/MS, was applied to perform routine analyses of diverse legume seeds. Eighteen samples of leguminous plants, including three samples of each type (field beans, peas, white lupins, yellow lupins, narrow-leaved lupins, lentils).

**Figure 3:**
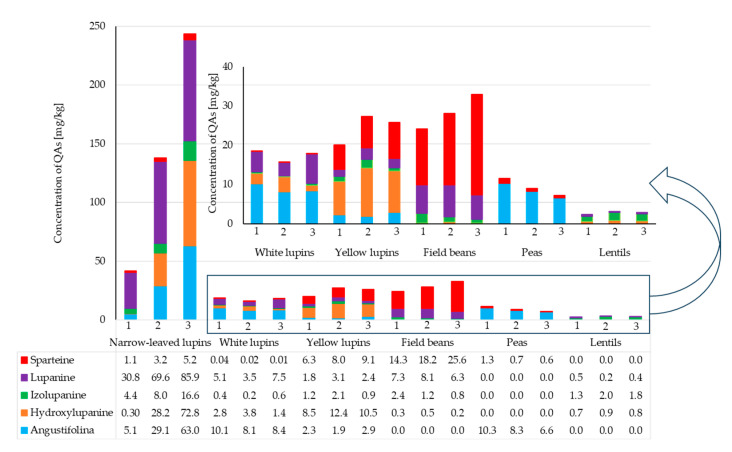
The presence of quinolizidine alkaloids in leguminous plants.

Samples of lentils had the lowest content of the five alkaloids tested, ranging from 2.4 to 2.9 mg/kg. In pea samples, the dominant compound was angustifoline (6.6–10.3 mg/kg) with significantly lower amounts of sparteine (0.58–1.25 mg/kg). Field bean seeds had the highest sparteine content (14.3–25.5 mg/kg). Narrow-leaved lupin and yellow lupin seeds contained higher concentrations of sparteine (1.3–9.1 mg/kg), in contrast to white lupin seeds (0.01–0.04 mg/kg). The most diverse composition of alkaloids was found in narrow-leaved lupin seeds. Five alkaloids were detected, with lupanine having the highest concentration (85.9 mg/kg) and sparteine having the lowest (1.1 mg/kg) (Figure 3, Supplementary Table S3). It was found that the diversity of alkaloids largely depends on the species of legume seeds studied, and each species has a unique alkaloid profile. Similarly to our study, research reports confirm the high content of lupanine, the most common alkaloid in white lupin [5,38,39], sparteine in yellow lupin, and lupanine, hydroxylupanine and angustifoline in narrow-leaved lupin [39].

Few literature reports confirm the presence of alkaloids; however, the results concern lupin samples [10,17]. Quantitative analyses demonstrated variation in the total content of five QAs in different lupin species, ranging from 3 to 31,800 mg/kg [10] and from 0.34 to 100 mg/kg [17]. The levels detected are similar to or significantly higher than those determined in our studies, which is due to the testing of different lupin species.

## 3. Materials and Methods

### 3.1. Materials

The test material consisted of samples of legume seeds: (1) field beans, (2) peas, (3) white lupins, (4) yellow lupins, (5) narrow-leaved lupins, and (6) lentils were collected from August to September 2024 in ecological local farm of Podlasie region, in the northeast of Poland. The samples were transferred frozen (on dry ice) to the Laboratory of Food and Feed Safety of the Institute of Plant Protection—National Institute of Poland. All legume seed samples (2 kg of samples) were homogenised and then mixed to ensure representativeness. All collected samples were stored at −20 °C until analysis. The samples were used for the initial development of the method and validation.

Analytical studies covered five quinolizidine alkaloids: angustifoline, hydroxylupanine, sparteine, and the geometric isomers isolupanine and lupanine (Table 2).

The preparation of samples for alkaloid content testing followed the QuEChERS technique [40]. Qualitative and quantitative determinations were performed using liquid chromatography with tandem mass spectrometry [41].

### 3.2. Chemicals and Reagents

Acetonitrile, methanol, formic acid, acetic acid, ethyl acetate (all LC–MS grade), and sodium hydroxide (NaOH) were obtained from Sigma-Aldrich (St. Louis, MO, USA). Pre-weighed QuEChERS kit containing analytical grade reagent: magnesium sulfate (MgSO_4_), sodium chloride (NaCl), trisodium citrate dihydrate (Na_3_C_6_H_5_O_7_·*2H_2_O or Na_3_Cit), disodium hydrogen citrate sesquehydrate (Na_2_HC_6_H_5_O_7_*1.5H_2_O or Na_2_Cit) salt were procured from Agilent Technologies (Santa Clara, CA, USA). Distilled water was produced by a Milli-Q purification system (Millipore, Bedford, MA, USA).

### 3.3. Standards

Analytical standards of QAs were supplied from Roth (Karlsruhe, Germany). Primary stock standard solutions were prepared separately at about 100 µg/mL concentration by dissolving an accurately weighed amount of the reference standard in methanol and stored at −20 °C. The working standard solutions of multiple compounds in concentrations of 0.01, 0.05, 0.1, 0.5, 1.0, 5.0 and 10.0 µg/mL were made by serial dissolving of the appropriate amounts of each stock solution in methanol and stored at about 4 °C.

### 3.4. Sample Preparation

An analytical portion of 1 g sample (narrow-leaved lupin, white lupin, yellow lupin, field bean, pea, lentil) was weighed into a 50 mL polypropylene tube (in the case of sample preparation for validation, enriched with reference mixtures). Then, 9 mL of water was added and left at room temperature for 15 min. The analytes were extracted with 10 mL of acetonitrile in the presence of a buffer solution: NaCl, MgSO_4_, Na_2_Cit/Na_3_Cit. The whole mixture was shaken on a Vortex shaker for 1 min, then placed in an ultrasonic bath for 10 min. Afterwards, 1 mL of 50% NaOH was added to the tube and shaken for an additional minute. Samples were centrifuged for 10 min at 4000 rpm. Then, 100 µL of supernatant was transferred to a 15 mL tube, and 4.9 mL of dilution solvent (methanol/water, 10:90 *v*/*v*) was added. One mL of sample extract was transferred to a vial by filtering through a 0.22 µm pore diameter filter.

### 3.5. LC–MS/MS Conditions

Qualitative and quantitative determinations were performed using an Exion LC AD liquid chromatograph coupled with a QTRAP 7500 tandem mass spectrometry (LC–MS/MS) system (AB Sciex Instruments, Foster City, CA, USA). The separation of analytes was performed using a KINETEX HILIC column (1.7 μm; 100 × 2.1 mm) maintained at a temperature of 40 °C. The volume of the injected sample was 10 µL. The mobile phase consisted of 0.1% formic acid in water (phase A) and 0.1% formic acid in acetonitrile (phase B). The analysis was carried out using gradient elution in the following programme: 0–0.5 min (A: 5%, B: 95%), 5–7.5 min (A: 95%, B: 5%), 8–10 min (A: 5%, B: 95%). Detection involved an electrospray ionisation (ESI) source in positive ion mode with the following parameters: needle voltage 4500 V, source temperature 450 °C, atomisation gas pressure 60 psi, auxiliary gas pressure 70 psi, and shielding gas pressure 35 psi. Nitrogen was used as a dispersant and collision gas. Table 3 presents the analysis parameters for the five QAs studied.

### 3.6. Method Validation

The developed method was validated according to SANTE guidelines provided by the European Commission, Directorate-General for Health and Food Safety [36].

In the validation process, blank samples (verified chromatographically) lupine (narrow-leaved, white, yellow), field beans, peas, and lentils were used. They were spiked with mixtures of standards at four concentration levels according to the procedure described above. The method was tested for linearity, deviation of the back-calculated concentration (DEV), recovery (R), matrix effect (ME), limit of quantification (LOQ), limit of detection (LOD), accuracy, precision and uncertainty (U). The validation parameters were determined and evaluated in accordance with the criteria contained in Table 4.

## 4. Conclusions

The study results indicate that the optimised QuEChERS method combined with LC–MS/MS can be successfully used for quantitative and qualitative determination of five quinolizidine alkaloids, including geometric isomers of lupanine and isolupanine, which are most commonly present in legume seeds. In addition, this quick and simple method allows for the analysis of alkaloids at very low levels (0.01 mg/kg) in different legume matrices is a key innovation of this study. Compared to other methods, this increased sensitivity provides a clear competitive advantage, especially in applications requiring precise monitoring of trace amounts. Satisfactory validation parameters at all stages of the analysis were obtained with 9 mL of water, acetonitrile as the extractant, citrate buffer, and 10 min ultrasound-assisted extraction. The highest efficiency of the system and good selectivity and specificity of QAs were observed with a 100 mm HILIC column, ensuring separation of the geometric isomers of lupanine and isolupanine, using water with 0.1% formic acid (phase A) and acetonitrile (phase B). The research procedure presented here is a valuable tool for studying differences in alkaloid content and profiles among various lupin varieties and legume species.

## Figures and Tables

**Table 1:** Validation parameters of the analysed quinolizidine alkaloids.

QA ^(1)^	0.01 (mg/kg)			0.1 (mg/kg)			1.0 (mg/kg)			10.0 (mg/kg)		
	R ^(2)^ (%) (RSD) ^(3)^	U ^(4)^ (%)	ME ^(5)^ (%)	R (%) (RSD)	U (%)	ME (%)	R (%) (RSD)	U (%)	ME (%)	R (%) (RSD)	U (%)	ME (%)
Narrow-leaved lupins												
A ^(6)^	84 (9)	26	1	83 (7)	18	−4	91 (4)	14	5	88 (2)	14	3
H ^(7)^	79 (3)	20	14	77 (3)	28	−18	86 (2)	18	12	86 (15)	15	11
I ^(8)^	72 (3)	17	−6	72 (7)	25	−9	82 (6)	11	−11	89 (7)	7	−13
L ^(9)^	115 (3)	16	12	98 (14)	18	−10	104 (9)	12	−11	95 (4)	7	−6
S ^(10)^	78 (4)	17	−10	71 (6)	17	−8	79 (4)	10	−11	74 (3)	1	−6
White lupins												
A	106 (4)	22	−19	104 (8)	19	−17	101 (3)	16	−15	106 (10)	9	−13
H	71 (4)	22	−14	73 (4)	16	−14	72 (1)	12	−14	79 (11)	23	−18
I	103 (2)	27	−14	94 (3)	21	−19	100 (5)	12	−13	99 (12)	6	−16
L	106 (3)	10	12	104 (5)	18	−10	101 (3)	12	10	105 (3)	5	−7
S	114 (2)	18	−13	104 (2)	18	−9	103 (6)	13	−9	99 (5)	9	−10
Yellow lupins												
A	112 (6)	19	−16	106 (8)	20	−15	107 (3)	14	−10	101 (6)	9	−12
H	92 (2)	20	−18	83 (3)	17	−15	85 (5)	14	−12	84 (2)	7	−11
I	79 (8)	22	−14	81 (9)	27	−12	80 (8)	20	−13	70 (8)	12	−13
L	74 (6)	20	13	78 (7)	23	−19	80 (8)	19	−17	72 (7)	15	−17
S	86 (7)	25	−8	84 (6)	28	−12	88 (6)	18	−7	93 (5)	14	−7
Peas												
A	83 (5)	20	−16	86 (7)	17	−9	87 (7)	16	−11	86 (8)	17	−11
H	84 (15)	28	−5	84 (5)	22	−13	98 (5)	21	−18	75 (8)	12	−20
I	79 (15)	27	9	84 (9)	24	−17	91 (8)	18	7	79 (5)	10	−7
L	72 (11)	23	−12	76 (7)	28	−14	78 (8)	14	−3	86 (6)	15	−3
S	73 (12)	25	−6	78 (8)	28	−20	77 (6)	23	−6	70 (9)	20	−6
Field beans												
A	92 (14)	25	−15	91 (5)	24	−19	87 (4)	17	−9	89 (8)	18	−9
H	77 (15)	27	−13	81 (8)	26	−6	84 (7)	13	−11	83 (4)	17	−11
I	77 (10)	23	−7	79 (3)	23	−9	84 (8)	18	−6	85 (8)	13	−6
L	76 (9)	19	−9	76 (5)	24	−14	74 (4)	17	−4	87 (9)	15	−12
S	71 (14)	20	−12	76 (9)	29	−13	77 (3)	22	−13	79 (8)	19	−19
Lentils												
A	85 (14)	22	−16	94 (8)	19	−7	90 (5)	24	−8	74 (10)	16	−12
H	82 (15)	27	−4	89 (6)	22	−12	96 (6)	18	−16	91 (7)	17	−9
I	71 (8)	23	−19	76 (7)	25	−7	83 (5)	18	−5	86 (6)	16	−13
L	74 (14)	24	−15	80 (6)	24	−12	87 (3)	14	−9	70 (9)	14	−17
S	76 (12)	26	−6	76 (9)	27	−6	84 (4)	16	−12	89 (8)	19	−14

^(1)^ QA—Quinolizidine alkaloids, ^(2)^ R—recovery, ^(3)^ RSD—relative standard deviation, ^(4)^ U—uncertainty, ^(5)^ ME—matrix effect, ^(6)^ A—Angustifolin, ^(7)^ H—Hydroxylupanine, ^(8)^ I—Izolupanine, ^(9)^ L—Lupanine, ^(10)^ S—Sparteine.

**Table 2:** Determined quinolizidine alkaloids (QAs).

QA (CAS Number)	Chemical Structure	Molecular Formula	Molecular Weight (g/mol)
(+)-Angustifoline (550-43-6)	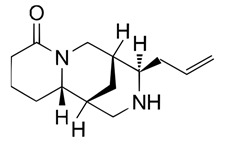	C_14_H_22_N_2_O	234.34
(+)-13α-Hydroxylupanine (15358-48-2)	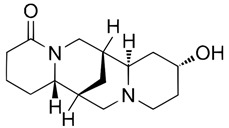	C_15_H_24_N_2_O_2_	264.36
α-Isolupanine (486-87-3)	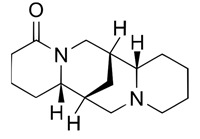	C_15_H_24_N_2_O	248.36
(+)-Lupanine (7400-11-5)	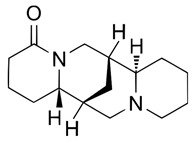	C_15_H_24_N_2_O	248.36
(−)-Sparteine (90-39-1)	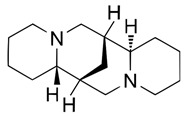	C_15_H_26_N_2_	234.39

**Table 3:** Acquisition parameters for the five quinolizidine alkaloids studied by LC-MS/MS.

Quinolizidine Alkaloids	Retention Time (min)	Precursor Ion (*m*/*z*)	DP ^(1)^/EP ^(2)^ (V)	Product Ion (*m*/*z*)	CE (V) ^(3)^	CXP (V) ^(4)^
Angustifolin	3.70	235.1	23/12	193/112.1 *	27/37	6/6
Hydroksylupanine	2.60	265	15/10	247.2/148.1 *	36/50	10/10
Izolupanine	3.55	248.9	21/11	136.1/114 *	41/38	4/8
Lupanine	3.30	248.9	21/11	136.1/114 *	41/38	4/8
Sparteine	4.95	235	32/10	98.1/233.1 *	49/38	8/8

^(1)^ DP—declustering potential, ^(2)^ EP—entrance potential, ^(3)^ CE—collision energy, ^(4)^ CXP—cell exit potential, * confirmation ion.

**Table 4:** Validation parameters and criteria.

Parameter	Criteria	How/What
Recovery	70–120% (30–140% in routine analyses)	Four spiking level (LOQ, 10 × LOQ, 100 × LOQ, 1000 × LOQ) in five replications.
RSD ^1^	≤20%	5 replications at each level (4 level).
Linearity	% DEV ^6^ = (C_measured_ − C_true_) × 100/C_true_) −20 ≤ DEV ≤ 20%	Matrix-matched calibration at 7 concentration levels (0.01, 0.05, 0.1, 0.5, 1.0, 5.0 and 10.0 µg/mL).
LOQ ^2^	S ^7^/N ^8^ ≥ 10	The lowest amount of the analyte in a sample that can be determined with acceptable precision and accuracy.
LOD ^3^	S/N ≥ 3	The lowest amount of analyte that can be detected but not necessarily quantified.
ME ^4^	% ME = (slope_matrix_/slopes_olvent_ − 1) × 100% –20% ≤ ME ≤20%	ME was estimated by comparing the slopes of seven points on matrix-matched and solvent calibration curves.
U ^5^	U ≤ 50%	U was calculated based on validation data using a “top-down” model with coverage factor k = 2 at a 95% confidence level.

^1^ RSD—Precision, ^2^ LOQ—Limit of quantification, ^3^ LOD—Limit of detection, ^4^ ME—Matrix effect, ^5^ U—Uncertainty, ^6^ DEV—deviation of the back-calculated concentration, ^7^ S—signal, ^8^ N—noise.

## Data Availability

Data is contained within the article.

## Supplementary Materials

The following supporting information can be downloaded at: https://www.mdpi.com/article/10.3390/molecules30204085/s1, Table S1: Optimisation of parameters for the preparation of alkaloids for LC-MS/MS analysis; Table S2: Influence of different parameters on the recovery (%); Table S3: Concentration (mg/kg) of quinolizidine alkaloids in leguminous plants.

## Abbreviations

The following abbreviations are used in this manuscript:

AOAC	Association of Official Analytical Chemists
C18	Octadecylsilane
DEV	Deviation of the Back-Calculated Concentration
EU	European Union
FID	Flame Ionization Detector
GC-MS	Gas Chromatography with Mass Spectrometry
LC-MS/MS	Liquid Chromatography Tandem Mass Spectrometry
LOD	Limit of Detection
LOQ	Limit of Quantification
MgSO_4_	Magnesium sulfate
ME	Matrix Effect
MRM	Multiple Reaction Monitoring
ML	Maximum Level
Na_2_HC_6_H_5_O_7_*1.5H_2_O	Disodium hydrogen citrate sesquehydrate
Na_3_C_6_H_5_O_7_*2H_2_O	Trisodium citrate dihydrate
NaCl	Sodium chloride
NaOH	Sodium hydroxide
PAs	Pyrrolizidine alkaloids
PAH	Polycyclic Aromatic Hydrocarbons
R	Recovery
S/N	Signal/Noise
SPE	Solid Phase Extraction
QuEChERS	Quick, Easy, Cheap, Effective, Rugged, and Safe
QAs	Quinolizidine Alkaloids
UHPLC-QTOF-MS	Ultra-High-Performance Liquid Chromatography-triple/time-of-flight Mass Spectrometry
TAs	Tropane alkaloids
U	Uncertainty

## References

[1] Dell’Olmo E., Tiberini A., Sigillo L. (2023). Leguminous Seedborne Pathogens: Seed Health and Sustainable Crop Management. Plants.

[2] Amarowicz R. (2020). Legume Seeds as an Important Component of Human Diet. Foods.

[3] Nwaji A.R., Arieri O., Anyang A.S., Nguedia K., Abiade E.B., Forcados G.E., Oladipo O.O., Makama S., Elisha I.L., Ozele N. (2022). Natural toxins and One Health: A review. Sci. One Health.

[4] Kishimoto S., Sato M., Tsunematsu Y., Watanabe K. (2016). Evaluation of Biosynthetic Pathway and Engineered Biosynthesis of Alkaloids. Molecules.

[5] Eugelio F., Palmieri S., Fanti F., Messuri L., Pepe A., Compagnone D., Sergi M. (2023). Development of an HPLC-MS/MS Method for the Determination of Alkaloids in Lupins. Molecules.

[6] Adaszyńska M., Swarcewicz M. (2013). Secondary plant metabolites as antimicrobial agents. Wiad. Chem..

[7] Kaczyński P., Łozowicka B. (2020). A novel approach for fast and simple determination pyrrolizidine alkaloids in herbs by ultrasound-assisted dispersive solid phase extraction method coupled to liquid chromatography-tandem mass spectrometry. J. Pharm. Biomed. Anal..

[8] Jankowska M., Łozowicka B. (2021). Natural and synthetic toxic substances occurring in agricultural plants and their products. Prog. Plant Prot..

[9] Wink M. (2019). Quinolizidine and pyrrolizidine alkaloid chemical ecology—A mini-review on their similarities and differences. J. Chem. Ecol..

[10] Namdar D., Mulder P.P.J., Ben-Simchon E., Hacham Y., Basheer L., Cohen O., Sternberg M., Shelef O. (2024). New Analytical Approach to Quinolizidine Alkaloids and Their Assumed Biosynthesis Pathways in Lupin Seeds. Toxins.

[11] Commission Regulation (EU) (2023). 2023/915 of 25 April 2023 on maximum levels for certain contaminants in food and repealing Regulation (EC) No 1881/2006. Off. J. Eur. Union.

[12] Hwang I.M., Lee H.W., Lee H.M., Yang J.S., Seo H.Y., Chung Y.J., Kim S.H. (2020). Rapid and Simultaneous Quantification of Five Quinolizidine Alkaloids in *Lupinus angustifolius* L. and Its Processed Foods by UPLC-MS/MS. ACS Omega.

[13] Otterbach S.L., Yang T., Kato L., Janfelt C., Geu-Flores F. (2019). Quinolizidine alkaloids are transported to seeds of bitter narrow-leafed lupin. J. Exp. Bot..

[14] Khedr T., Juhász A., Singh K.B., Foley R., Nye-Wood M.G., Colgrave M.L. (2023). Development and validation of a rapid and sensitive LC-MS/MS approach for alkaloid testing in different *Lupinus* species. J. Food Compos. Anal..

[15] Roman L., Tsochatzis E., Tarin K., Röndahl E.M., Ottosen C.-O., Corredig M. (2023). Compositional Attributes of Blue Lupin (*Lupinus angustifolius*) Seeds for Selection of High-Protein Cultivars. J. Agric. Food Chem..

[16] Cortés-Avendaño P., Tarvainen M., Suomela J.P., Glorio-Paulet P., Yang B., Repo-Carrasco-Valencia R. (2020). Profile and Content of Residual Alkaloids in Ten Ecotypes of *Lupinus mutabilis* Sweet after Aqueous Debittering Process. Plant Foods Hum. Nutr..

[17] Święcicki W., Czepiel K., Wilczura P., Barzyk P., Kaczmarek Z., Kroc M. (2019). Chromatographic Fingerprinting of the Old World Lupins Seed Alkaloids: A Supplemental Tool in Species Discrimination. Plants.

[18] Anastassiades M., Lehotay S.J., Stajnbaher D., Schenck F.J. (2003). Fast and easy multiresidue method employing acetonitrile extraction/partitioning and dispersive soildphase extraction for the determination of pesticide residues in produce. J. AOAC Int..

[19] Łozowicka B., Kaczyński P., Jankowska M., Rutkowska E., Iwaniuk P., Konecki R., Rogowska W., Zhagyparova A., Absatarova D., Łuniewski S. (2025). Evaluation of Broad-Spectrum Pesticides Based on Unified Multi-Analytical Procedure in Fruits and Vegetables for Acute Health Risk Assessment. Foods.

[20] Hrynko I., Kaczyński P., Łozowicka B. (2021). A global study of pesticides in bees: QuEChERS as a sample preparation methodology for their analysis—Critical review and perspective. Sci. Total Environ..

[21] García-Vara M., Postigo C., Palma P., Bleda M.J., de Alda M.L. (2022). QuEChERS-based analytical methods developed for LC-MS/MS multiresidue determination of pesticides in representative crop fatty matrices: Olives and sunflower seeds. Food Chem..

[22] Hakami R.A., Aqel A., Ghfar A.A., ALOthman Z.A., Badjah-Hadj-Ahmed A.-Y. (2021). Development of QuEChERS extraction method for the determination of pesticide residues in cereals using DART-ToF-MS and GC-MS techniques. Correlation and quantification study. J. Compos. Anal..

[23] Kaczyński P., Iwaniuk P., Jankowska M., Orywal K., Socha K., Perkowski M., Ali Farhan J., Łozowicka B. (2024). Pesticide residues in common and herbal teas combined with risk assessment and transfer to the infusiom. Chemosphere.

[24] Wang J., Yang H.-Y., Wang X.-D., Lv Y.-F., Wei N. (2025). Application of QuEChERS for Analysis of Contaminants in Dairy Products: A Review. J. Food Prot..

[25] Piechowicz B., Kuliga A., Kobylarz D., Koziorowska A., Zaręba L., Podbielska M., Piechowicz I., and Sadło S. (2022). A case study on the occurrence of pyrimethanil, cyprodinil and cyflufenamid residues in soil and on apple leaves, blossoms and pollen, and their transfer by worker bees to the hive. J. Plant Prot. Res..

[26] Sadighara P., Basaran B., Afshar A., Nazmara S. (2024). Optimization of clean-up in QuEChERS method for extraction of mycotoxins in food samples: A systematic review. Microchem. J..

[27] Ingegno M., Zianni R., Della Rovere I., Chiappinelli A., Nardelli V., Casamassima F.D., Calitri A., Quinto M., Nardiello D., Iammarino M. (2024). Development of a highly sensitive method based on QuEChERS and GC–MS/MS for the determination of polycyclic aromatic hydrocarbons in infant foods. Front. Nutr..

[28] Rivai S.N., Kristianto S., Putri R.E., Fatiqin A., Abidin M.H.Z. (2025). Modification of the QuEChERS Method for Drug Analysis in Biological Sample: A Review. J. Multidiscip. Appl. Nat. Sci..

[29] Wang H., Xu X., Wang X., Guo W., Jia W., Zhang F. (2022). An analytical strategy for discovering structural analogues of alkaloids in plant food using characteristic structural fragments extraction by high resolution orbitrap mass spectrometry. LWT-Food Sci. Technol..

[30] Buszewski B., Noga S. (2012). Hydrophilic interaction liquid chromatography (HILIC)—A powerful separation technique. Anal. Bioanal. Chem..

[31] AOAC International (2007). AOAC Official Method 2007.01, Pesticide Residues in Foods by Acetonitrile Extraction and Partitioning with Magnesium Sulfate: Gas Chromatography/Mass Spectrometry and Liquid Chromatography/Tandem Mass Spectrometry First Action 2007.

[32] (2018). Foods of Plant origin—Multimethod for the Determination of Pesticide Residues Using GC and/or LC-based Analysis Following Acetonitrile Extraction/Partioning and Clean-up by Dispersive SPE–Modular QuEChERS-Method.

[33] Čajka T., Sandy C., Bachanova V., Drabova L., Kalachova K., Pulkrabova J., Hajšlová J. (2012). Streamlining sample preparation and gas chromatography-tandem mass spectrometry analysis of multiple pesticide residues in tea. Anal. Chim. Acta.

[34] Perestrelo R., Silva P., Porto-Figueira P., Pereira J.A.M., Silva C., Medina S., Câmara J.S. (2019). QuEChERS—Fundamentals, relevant improvements, applications and future trends. Anal. Chim. Acta.

[35] García-Valcárcel A.I., Martínez-Ferrer M.T., Campos-Rivela J.M., Guil M.D.H. (2019). Analysis of pesticide residues in honeybee (*Apis mellifera* L.) and in corbicular pollen. Exposure in citrus orchard with an integrated pest management system. Talanta.

[36] SANTE Guidance Document on Analytical Quality Control and Method Validation Procedures for Pesticide Residues Analysis in Food and Feed. SANTE/11312/2021, 2021, 1–51. https://food.ec.europa.eu/system/files/2023-11/pesticides_mrl_guidelines_wrkdoc_2021-11312.pdf.

[37] Remane D., Meyer M.R., Wissenbach D.K., Maurer H.H. (2010). Ion suppression and enhancement effects of co-eluting analytes in multi-analyte approaches: Systematic investigation using ultra-high-performance liquid chromatography/mass spectrometry with atmospheric-pressure chemical ionization or electrospray ionization. Rapid Commun. Mass Spectrom..

[38] Kroc M., Rybiński W., Wilczura P., Kamel K., Kaczmarek Z., Barzyk P., Święcicki W. (2017). Quantitative and qualitative analysis of alkaloids composition in the seeds of a white lupin (*Lupinus albus* L.) collection. Genet. Resour. Crop Evol..

[39] Romeo F.V., Fabroni S., Ballistreri G., Muccilli S., Spina A., Rapisarda P. (2018). Characterization and antimicrobial activity of alkaloid extracts from seeds of different genotypes of *Lupinus* spp.. Sustainability.

[40] Rutkowska E., Kaczyński P., Iwaniuk P., Łozowicka B., Hrynko I., Jankowska M., Konecki R., Rogowska W., Rusiłowska J., Pietkun M. (2025). An extensive pesticide residue study in minor polish vegetables based on critical consumer diets. Food Control.

[41] Iwaniuk P., Łuniewski S., Kaczyński P., Łozowicka B. (2023). The Influence of Humic Acids and Nitrophenols on Metabolic Compounds and Pesticide Behavior in Wheat under Biotic Stress. Agronomy.

